# Prediction of Renal Acid Load in Adult Patients on Parenteral Nutrition

**DOI:** 10.3390/pharmaceutics10020043

**Published:** 2018-04-02

**Authors:** Roberto Iacone, Clelia Scanzano, Anna D’Isanto, Andrea Vitalone, Ignazio Frangipane, Mariana D’Angeli, Lidia Santarpia, Franco Contaldo

**Affiliations:** Clinical Nutrition Unit—Department of Clinical Medicine and Surgery, “Federico II” University Medical School, via S. Pansini 5, 80131 Naples, Italy; clelia.scanzano@unina.it (C.S.); annadisanto85@hotmail.it (A.D.); andreavitalone1989@gmail.com (A.V.); frangipane.ignazio@gmail.com (I.F.); dangelimariana@outlook.it (M.D.); lidia.santarpia@unina.it (L.S.); franco.contaldo@unina.it (F.C.)

**Keywords:** parenteral nutrition, total renal acid load, metabolic renal acid load, pH, titratable acidity

## Abstract

Metabolic acidosis and metabolic bone disease are frequent complications in patients on parenteral nutrition (PN). A common contributor to these complications could be a daily high renal acid load. This study aims to find a method for predicting the potential total acid load (PTAL) and the pH of the compounded parenteral nutrition mixtures. The pH and titratable acidity (TA) of fifty compounded mixtures were measured. The potential metabolic acid load (PMAL) was calculated by considering the amount of nutrients that are acid producers and consumers. The PTAL of the TPN mixtures was calculated by adding TA to PMAL. Multiple linear regression analyses were used to develop a predictive model for the TA and pH of the compounded mixtures. The predicted TA and pH values of the analyzed mixtures agreed with those measured (Passing-Bablok analysis). The PTAL was >50 mmol/day for 82% of the mixtures, >75 mmol/day for 40% of the mixtures, and >100 mmol/day for 22% of the mixtures. The prediction of the renal acid load in patients on long-term PN could allow more appropriate acid-base balancing. Moreover, predicting the pH of such mixtures could be useful to pharmacists to assess the stability and compatibility of the components in the compounded mixtures.

## 1. Introduction

Parenteral nutrition (PN) may be associated with several metabolic complications including metabolic acidosis (MA) and metabolic bone disease (MBD). MA during PN may be caused by the excessive exogenous administration and/or endogenous production of non-volatile acids or by a reduction in the renal excretion of fixed acid or massive loss of bicarbonate through the gastrointestinal tract. In PN patients, MA is an under-reported metabolic complication [1] and data on the prevalence of metabolic disturbances in PN patients are not still comprehensively reported and, depending on the type of study, ranged from 5% to 25% [1,2]. MBD has a greater prevalence in patients on long-term artificial nutrition than in the general population with a particularly high prevalence (67–84%) in patients on long-term PN [3,4]. However, the etiology of MBD in patients on long-term PN is not well-known [5,6]. Studies indicate that one of the possible causes of this complication could depend on a chronic, not excessive, renal acid load following daily administration of PN mixtures [7,8]. The intravenous infusion of these mixtures is associated both with already formed non-volatile acids (titratable acidity, TA) [2,9] and non-volatile acids resulting from the catabolism of some nutrients (potential metabolic acid load, PMAL). As a consequence, a persistent and imbalanced daily renal acid load would, therefore, represent a common factor among PN patients with overt MBD. Although it is still being debated, one hypothesis [10] that is sometimes confirmed or contradicted [11] describes that a constant renal acid load triggers the release of alkaline substances from the bone, which are mainly calcium and carbonates. This is a physiological process that may help the body maintain the pH of the blood at acceptable biological levels. On the other hand, this would weaken the bone mineral matrix by causing osteopenia and then osteoporosis over time. In patients exclusively receiving PN, the renal acid load depends entirely on the qualitative and quantitative composition of the compounded mixture used for parenteral nutrition. The prediction of the potential total acid load (PTAL) of the mixture could allow the nutrition specialist to balance the components of the mixture to obtain a very low or nil PTAL. The primary purpose of this study was to develop a predictive equation for the PTAL based on the content of all components in a compounded PN mixture. The secondary aim of the study was to then develop an equation for predicting the pH. The pH of the compounded mixtures for PN does not play a role in the evaluation of the PTAL because the free acidity (pH) is already included in the TA and a compounded mixture may have a relatively low pH but a very low, none, or negative PTAL. However, having the knowledge of the compounded mixture’s pH in advance is important because pH is the leading factor used for predicting the stability and compatibility of the components of such mixtures.

## 2. Materials and Methods

Fifty patients receiving individualized compounded mixtures of PN were evaluated by the Clinical Nutrition Unit of Federico II University Medical School. The compounding of PN mixtures was carried out using an automatic filler (SIFRAMIX filling system, Fresenius Kabi Italia, Isola della Scala, Italy) in which the nutrients in the bag were added in the following order of processing: water, glucose, and amino acids simultaneously. The order of addition of electrolytes was first sodium and then potassium, magnesium, calcium, and 1,6-diphosphate. Lipid was added at the same time of the other macronutrients but in a separate compartment of the bag. The nutrients used to compound the mixtures were as follows: 10% Sintamin (5000 mL bag, Fresenius Kabi Italia, Isola della Scala, Italy), 50% glucose (5000 mL bag, GalenicaSenese, Monteroni d’Arbia, Italy), 20% Smoflipid (500 mL bottle, Fresenius Kabi Italia, Isola della Scala, Italy), 6% gluconate calcium (250 mL bottle, Bioindustria, Novi Ligure, Italy), 0.5 mmol/mL magnesium sulfate (250 mL bottle, Bioindustria, Novi Ligure, Italy), 10 g/100 mL D-fructose 1,6-diphosphate disodium salt hydrate (100 mL bottle, Biomedica Foscama Group Spa, Ferentino, Italy), 3 mmol/mL sodium chloride (250 mL bottle, Bioindustria, Novi Ligure, Italy), 3 mmol/mL potassium chloride (250 mL bottle, Fresenius Kabi Italia, Isola della Scala, Italy), and water for injections (5000 mL bag, GalenicaSenese, Monteroni d’Arbia, Italy). We recommend adding vitamins (Soluvit and Vitalipid) and trace elements (Addamel N) to the PN mixtures immediately before the infusion. The pH of the mixtures was measured by using a Beckman Φ40 pHmeter, which was calibrated with three standards at pH values of 4, 7, and 10. TA was measured in millimoles by adding 0.1 M NaOH until pH equaled 7.4. The pH and TA measurements of the tested PN mixtures were made in duplicate at room temperature immediately after compounding and after subsequent storage for one day at room temperature. This is roughly the time needed for infusion. According to the standard method, a precision of ±0.02 pH unit and an accuracy of ±0.05 pH unit was accepted. The pH values are reported to the nearest 0.01 pH units. The TA values are reported to the nearest 0.1 mmol units. PMAL was calculated by summing the millimoles of non-volatile acid producers (cysteine, methionine, lysine, histidine, arginine, and phospholipids) and by subtracting the millimoles of fixed acid consumers (acetate and gluconate) included in the analyzed mixtures. The PMAL of compounded mixtures prescribed for PN patients was calculated using the formula below.

PMAL (mmol) = [(2 × cysteine) + (2 × methionine) + lysine + histidine + arginine + (phospholipids/5) − acetate − gluconate] × (volume/1000).

Cysteine, methionine, lysine, histidine, arginine, phospholipids, acetate, and gluconate are expressed in mM and volume is expressed in ml. The anionic amino acids (aspartate and glutamate) were not considered in the calculation because they were not present in the amino acid solution (10% Sintamin) used for the compounding of patient-specific mixtures. The anionic acetate was present in the amino acid solution as stabilizing (85 mmol/L) and the anionic gluconate derived from the calcium gluconate. The phospholipids were from the lipid emulsion, 15 mmol/L (20% Smoflipid). The PTAL of the PN mixtures was calculated as the algebraic sum of the TA and the PMAL.

## 3. Statistics

Multiple linear regression analyses (based on the quantitative and qualitative composition of the analyzed mixtures and on pH and TA measurements) were used to develop a predictive model for pH and TA. pH and TA measurements of the PN mixtures were carried out after mixing for each preparation. This included the prescribed doses of all components. The agreement between the experimental results and those provided by the multilinear model was evaluated by the Passing-Bablok non-parametric regression analysis and by the Bland-Altman plot. Two-sided *p* values less than 0.05 were considered statistically significant.

## 4. Results

Table 1 shows the mean, the standard deviation, and the minimum-maximum concentrations of the components utilized in the compounding of the evaluated parenteral nutrition mixtures.

### 4.1. Predictive Equations for the Titratable Acidity and the pH of TPN Mixtures

The amount of the nutrients added to each mixture was used for creating predictive equations for TA and pH. Multiple regression analyses (method enter: dependent variable = measured TA, independent variables = concentration of all constituents of the mixture; r = 0.998, *p* < 0.0001) generated the equation below.

TA_predicted_ (mmol) = [0.769 + (−0.029 × glucose) + (0.619 × lipid) + (1.001 × amino acid) + (0.078 × calcium) + (0.487 × phosphorous) + (−0.004 × sodium) + (0.002 × potassium) + (−0.070 × magnesium)] × (volume/1000).

When using the measured pH (r = 0.959, *p* < 0.0001) as the dependent variable, the equationbelow was obtained.

pH_predicted_ = 5.894 + (−0.007 × glucose) + (0.026 × lipid) + (0.050 × amino acid) + (0.009 × calcium) + (−0.036 × phosphorous) + (−0.001 × sodium) + (0.0003 × potassium) + (−0.003 × magnesium).

These two predictive equations led to the prediction of the total titratable acidity (TA_predicted_) and the pH (pH_predicted_) of the compounded mixtures, according to the content of their constituents.

Glucose, lipids, and amino acids are expressed in g/100 mL. Calcium (as calcium gluconate), phosphorus (as D-fructose 1,6-diphosphate disodium salt hydrate), sodium (as sodium chloride), potassium (as potassium chloride), and magnesium (as magnesium sulfate) are expressed in mM.

### 4.2. Comparison of Measured and Predicted Titratable Acidity and pH

**Figure 1:**
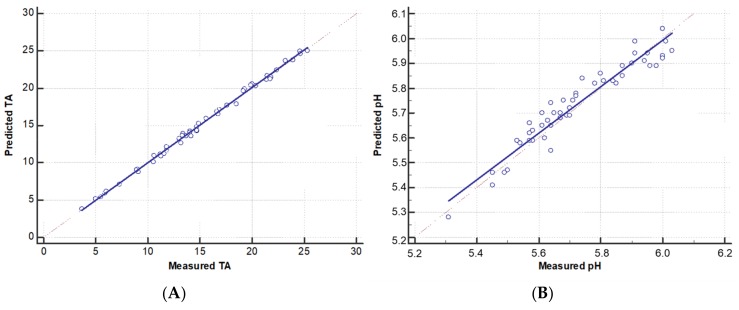
Scatter-plot with the regression line (calculated according to the Passing-Bablok method) and the identity line for the measured and predicted values of titratable acidity (TA), (**A**) and pH (**B**). TA is expressed in millimoles of NaOH added until pH = 7.4.

**Figure 2:**
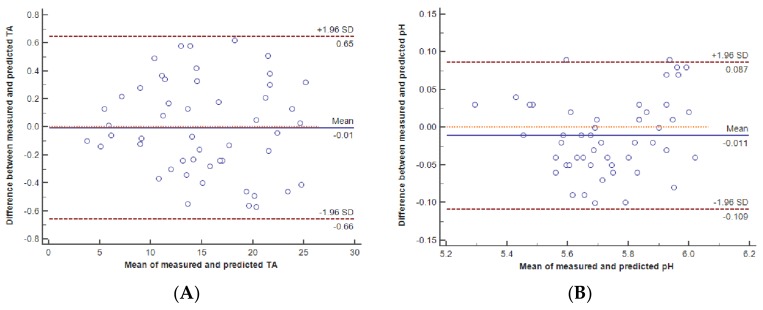
Bland-Altman plots for the measured and predicted values of titratable acidity (TA), (**A**) and pH (**B**).

Measured and predicted TA and pH values of the evaluated mixtures were normally distributed (Kolmogorov-Smirnorv test, *p* = 0.20). The mean, 95% confidence interval, and minimum and maximum of the measured and predicted TA and pH are shown in Table 2. Figure 1A,B depict the scatter-plot with the trend and the identity lines between the measured and predicted values of TA and pH, respectively. The cusum test for the linear model of the measured and predicted values did not show significant deviations from linearity for TA (*p* = 0.44) or pH (*p* = 0.26) and allowed the application of the Passing-Bablok regression. The Passing-Bablok regression equation for measured and predicted TA was as follows: Y = 1.001X − 0.040. The 95% confidence intervals (95% CIs) for the slope and the intercept were 0.987 to 1.024 and −0.281 to 0.201, respectively. The Passing-Bablok regression equation for the measured and predicted pH was as follows: Y = 0.939X + 0.360; 95% CI = 0.842 to 1.035 for the slope and 95% CI= −0.181 to 0.923 for the intercept. For both equations, the 95% CI for the trend line coefficients contained 0 for the intercept and 1 for the slope, which validated the congruity between the measured and predicted values for both TA and pH. Figure 2A,B show the Bland-Altman plots for the measured and predicted values of TA and pH, respectively. In both graphs, the points are scattered within the two 95% CIs for the means of the differences, which shows that the predicted results were in agreement with the measured ones. Vitamins and trace elements didn’t significantly alter the pH or TA of the PN mixture. Typically, the pH and TA values of a mixture before and after addition of vitamins and trace elements were at a pH 5.98–6.01 and a TA 18.33–18.37 mmol of NaOH, respectively.

### 4.3. Calculation of the Potential Metabolic Acid Load

Table 3 shows the mean, the standard deviation, and the minimum–maximum concentration of producers and consumers of non-volatile acids after their metabolization.

### 4.4. Calculation of the Potential Total Acid Load

The PTAL of the TPN mixtures was calculated using the following equation: PTAL = TA + PMAL. Table 4 shows the mean, the standard deviation, and the minimum and maximum of TA, PMAL, and PTAL. The data showed that, on average, approximately 80% of the PTAL depended on the PMAL and the remaining percentage was from the preformed non-volatile acids. The PTAL values of the fifty considered mixtures were between approximately 5 and 152 mmol/day. Regarding the distribution of these values, the PTAL was >50 mmol/day in the 82% of the mixtures (41/50), >75 mmol/day in 40% (20/50), and >100 mmol/day in 22% of the mixtures (11/50).

## 5. Discussion

A risk of metabolic acidosis associated with PN in patients without renal dysfunction or bicarbonate loss is clearly correlated with an excessive and acute renal acid load that could be due to the content of both preformed non-volatile acids and non-volatile acids that result from the catabolism of nutrients present in the administered mixture [12]. The preformed non-volatile acids originate from the chlorine, phosphate, or sulfate anions of PN mixture components or from the inorganic acids added to the single solutions of macronutrients (lipid emulsion, amino acid mixture, and glucose) and electrolytes to maintain their stability over time or to reach an advantageous pH for the physical-chemical stability of the compounded mixture after their mixing. The fixed acidity produced by the catabolism of nutrients when compared to energy is called “potential” because it depends on the amino acids’ metabolic fate. In patients with a nil nitrogen balance where the body protein content remains constant, the amount of amino acids administered equals that of protein turnover. Since the amino acids from protein turnover are all sent to the oxidative degradation pathway, the production of non-volatile acids is equal to the amount of amino acids acid producers provided with the mixtures. In patients with a positive or negative nitrogen balance, the metabolic production of non-volatile acids could be reduced or increased compared to the amino acid content administered in the parenteral nutrition mixtures. Our results show that the non-volatile acid producers compared to the preformed non-volatile acids considerably affect (80% on average) the PTAL of the compounded PN mixtures. Studies report that the MA is mainly caused by the addition of preformed inorganic acids to adjust the pH in a favorable range and improve the stability of the compounded PN mixture [2,3,4,5,6,7,8,9] rather than the acidity of metabolic origin. In any case, it is reasonable to assume that, when the daily PTAL significantly exceeds the capacity of body buffer systems in the event of impaired renal function or when an excessive loss of bicarbonate occurs, it is highly probable that acute MA may occur if the infusion of a mixture with an elevated PTAL is performed at high speed. This is a relevant clinical event with the pathological reduction of pH, bicarbonates, and the partial pressure of carbon dioxide in the arterial blood, which generally requires a timely medical intervention. On the other hand, chronic MA with secondary osteopenia or osteoporosis shows a slight reduction in the arterial blood pH, which could still be within the values considered normal [13]. MBD associated with long-term PN has an etiology not still fully explained and is likely related to several persistent factors such as chronic malnutrition, multiple vitamin and mineral deficiencies, drug use such as corticosteroids, contaminants present in the solutions used for the compounding of PN mixtures (e.g., aluminum), and underlying pathologies [5,6]. What is certain is that MBD is common in long-term PN patients and is characterized by initially asymptomatic osteopenia/osteoporosis that becomes evident over time and includes bone pain and increased risk of fractures. It has been suggested that MBD may be due to a silent chronic acidemia [13,14] that would activate a slow but constant release of alkaline substances from the bone to balance the daily acid load. By this mechanism, the HCO_3_^¯^ and pH values in the blood would still fall within the limits considered “normal” but would be slightly below the ideal value. This would help maintain the activity of the stimuli (as an increase in renal excretion of NH_4_^+^, H^+^, and H_2_PO_4_^−^ ions or renal generation/recovery of bicarbonate ions) for the homeostatic adaptations necessary to achieve the appropriate acid-base balance. When the renal mechanisms involved in acid-base balance are insufficient to counterbalance a high and persistent PTAL, a release of alkaline substances from the bone occurs, which helps to maintain the blood pH at acceptable levels for the body but at the cost of bone mineral depletion over time.

The results of the present study show that a significant percentage of patients on PN are exposed daily to a potential renal acid load greater than 50 mmol. In several cases, the potential renal acid load exceeds 100 mmol/day, which predisposes patients to MA or possible metabolic complications of the bone. As a consequence, it is evident that the prediction of the daily PTAL in long-term PN patients becomes essential for the prevention of these complications. The use of a simple equation for predicting the acid load related to the quality and quantity of constituents coupled with the simple calculation of the PMAL resulting from the nutrients’ metabolism used for PN could be a useful tool available to the nutrition specialist to balance the PTAL of the mixtures for infusion. If feasible, the PTAL of the mixture could be corrected by adding the right dose of organic acids or salts of organic acids such as acetate, gluconate, aspartate, or citrate without interfering significantly on the final pH of the compounded mixture. Otherwise, the nutrition specialist could provide an additional parenteral administration separately from the PN mixture of alkalinizing solutions (e.g., potassium lactate or sodium bicarbonate) to balance the daily acid load following the administration of the PN mixture. This would be of particular utility in long-term PN patients. Therefore, it could be recommended to pharmacists to report the daily PTAL on the label of the compounded mixture together with the information on the added nutrients. The authors can provide only general hints about how to balance the acid load due to the mixture infusion. The choice to change the type of nutrient used, e.g., potassium aspartate or acetate instead of potassium chloride, must be agreed between pharmacist and nutrition specialist since the anions of organic acids may not be tolerated by the patient or could affect the stability of the mixture. Therefore, it will be based on the pharmacist’s experience (to evaluate the possibility of a direct acid-base balance of the mixtures) and the clinical evaluation of the patient by the nutrition specialist that will be taken into consideration on a case-by-case basis.

Furthermore, predicting the pH of the mixture for PN based on the quantity and quality of its components could be very useful to the pharmacist for checking the stability and compatibility of the mixture constituents. A pH between 5.0 and 5.5 generally ensures an unsuitable environment for the precipitation of calcium phosphates [15], prevents the formation of toxic chemical species from the reaction between glucose and amino acids (Maillard reaction) [16,17], and discourages the aggregation and coalescence of the micelles present in the lipid emulsion when they enter into contact with the other components of the mixture [18]. Obviously, the predictive equations for the PTAL and pH of the compounded mixtures depend not only on the amount but also on the type of nutrients and electrolytes present in the utilized solutions. A wide range of electrolytes and macronutrients are commercially available and various pharmaceutical companies use different acidification methods to stabilize such solutions. Consequently, for each set of new products, it is necessary to perform a number of acid-base titrations on the compounded mixtures to establish, with a good margin of accuracy, the predictive equations for both PTAL and pH.

## 6. Conclusions

This study suggests a method for predicting the pH and the potential renal acid load of compounded parenteral nutrition mixtures to which adult long-term PN patients are exposed to daily. The prediction of the renal acid load may be of great utility to nutrition specialists since the knowledge of this value would allow a most appropriate acid-base balancing that would avoid or reduce some metabolic complications associated with long-term PN. Regarding PN patients who are in mixed nutrition such as parenteral and enteral nutrition or diet or oral nutritional supplements, the nutrition specialist will evaluate the additional acid load from sources other than the PN. Furthermore, although the pH of such mixtures does not affect the renal acid load, it could be useful to pharmacists in predicting the stability and compatibility of the many components added in the compounded mixtures.

## Figures and Tables

**Table 1:** Mean ± SD and minimum-maximum (min–max) values of the volume, osmolarity, and concentration of components in the compounded parenteral mixtures evaluated in thestudy (*n* = 50).

	Mean ± SD	Min–Max
Volume (mL)	1979 ± 574	630–3000
Osmolarity (mOsm/L)	1013 ± 236	419–1515
Glucose (g/100 mL)	9.1 ± 3.1	3.0–21.1
Lipid (g/100 mL)	2.2 ± 0.7	0–3.6
Amino Acid (g/100 mL))	3.3 ± 1.2	1.1–6.1
Calcium (mmol/L)	3.0 ± 2.2	0–14.3
Phosphorous (mmol/L)	6.1 ± 4.4	0–18.6
Sodium (mmol/L)	51.1 ± 18.0	15.0–90.0
Potassium (mmol/L)	25.2 ± 13.4	0–73.4
Magnesium (mmol/L)	4.6 ± 2.7	1.2–13.2
Acetate (mmol/L)	29.1 ± 10.5	9.6–53.7
Gluconate (mmol/mL)	5.8 ± 4.1	0–27.1
Chloride (mmol/mL)	76.3 ± 25.6	31.4–140.0

**Table 2:** Mean, 95% confidence interval (95% CI), and minimum-maximum (min–max) values of the measured and predicted titratable acidity (TA) and pH of compounded parenteral mixtures evaluated in this study (*n* = 50).

	Mean (95% CI)	Min–Max
Measured TA (mmol)	15.2 (13.6–16.8)	3.7–25.3
Predicted TA (mmol)	15.2 (13.6–16.8)	3.8–25.0
Measured pH	5.73 (5.68–5.78)	5.31–6.03
Predicted pH	5.74 (5.69–5.79)	5.28–6.04

**Table 3:** Mean ± SD and minimum-maximum (min–max) values of the concentration of substances in the compounded parenteral mixtures evaluated in this study (*n* = 50) that affect the renal acid load after their metabolism.

	Mean ± SD	Min–Max
Cysteine (mmol/L)	0.4 ± 01	0.1–0.7
Methionine (mmol/L)	12.0 ± 4.3	4.0–22.2
Lysine (mmol/L)	17.0 ± 6.1	5.6–31.4
Arginine (mmol/L)	18.5 ± 6.7	6.1–34.2
Histhidine (mmol/L)	7.2 ± 2.6	2.4–13.4
Phospholipid (mmol/L)	1.5 ± 0.5	0–2.6
Acetate (mmol/L)	29.1 ± 10.5	9.6–53.7
Gluconate (mmol/L)	5.8 ± 4.1	0–27.1

**Table 4:** Mean ± SD and minimum-maximum (min–max) values of the measured titratable acidity (TA), the calculated potential metabolic acid load (PMAL), and the potential total acid load (PTAL) in the compounded parenteral mixtures evaluated in this study (*n* = 50).

	Mean ± SD	Min–Max
TA (mmol)	15.2 ± 5.7	3.7–25.3
PMAL (mmol)	65.1 ± 28.5	−0.6–131.9
PTAL (mmol)	80.3 ± 32.6	5.3–151.8

## Abbreviations

MA	Metabolic acidosis
MBD	Metabolic bone disease
PMAL	Potential metabolic acid load
PTAL	Potential total acid load
TA	Titratable acidity
PN	Parenteral nutrition
